# Accelerated sonothrombolysis with Definity in a xenographic porcine cerebral thromboembolism model

**DOI:** 10.1038/s41598-021-83442-3

**Published:** 2021-02-17

**Authors:** Robert T. Kleven, Kunal B. Karani, Nicole Hilvert, Samantha M. Ford, Karla P. Mercado-Shekhar, John M. Racadio, Marepalli B. Rao, Todd A. Abruzzo, Christy K. Holland

**Affiliations:** 1 Department of Biomedical Engineering, College of Engineering and Applied Sciences, University of Cincinnati, CVC 3921, 0586, 231 Albert Sabin Way, Cincinnati, OH 45267-0586 USA; 2 Department of Radiology and Medical Imaging, Cincinnati Children’s Hospital Medical Center, Cincinnati, OH USA; 3 Department of Internal Medicine, Division of Cardiovascular Health and Disease, University of Cincinnati, Cincinnati, OH USA; 4 Division of Radiology, Phoenix Children’s Hospital, Phoenix, AZ USA; 5 Department of Radiology, University of Arizona College of Medicine, Phoenix, AZ USA

**Keywords:** Medical research, Neurology, Engineering

## Abstract

Adjuvant ultrasound at 2 MHz with or without an ultrasound contrast agent improves the rate of thrombus resolution by recombinant tissue plasminogen activator (rt-PA) in laboratory and clinical studies. A sub-megahertz approach can further expand this therapy to a subset of patients with an insufficient temporal bone window, improving efficacy in unselected patient populations. The aim of this study was to determine if a clinical ultrasound contrast agent (UCA), Definity, and 220 kHz pulsed ultrasound accelerated rt-PA thrombolysis in a preclinical animal model of vascular occlusion. The effect of Definity and ultrasound on thrombus clearance was first investigated in vitro and subsequently tested in a xenographic porcine cerebral thromboembolism model in vivo. Two different microcatheter designs (end-hole, multi-side-hole) were used to infuse rt-PA and Definity at the proximal edge or directly into clots, respectively. Sonothrombolysis with Definity increased clot mass loss relative to saline or rt-PA alone in vitro, only when rt-PA was administered directly into clots via a multi-side-hole microcatheter. Combined treatment with rt-PA, Definity, and ultrasound in vivo increased the rate of reperfusion up to 45 min faster than clots treated with rt-PA or saline. In this porcine cerebral thromboembolism model employing retracted human clots, 220 kHz ultrasound, in conjunction with Definity increased the probability of early successful reperfusion with rt-PA.

## Introduction

Sonothrombolysis has been explored as an adjuvant therapy for the treatment of ischemic stroke^1–3^. Although technical efficacy of sonothrombolysis has been demonstrated in the laboratory^3^, this approach has produced equivocal results in acute stroke trials using focused 2 MHz transcranial Doppler (TCD) ultrasound^4–8^. Laboratory and clinical studies have established that exogenous cavitation nuclei can further enhance and accelerate sonothrombolysis^3,4,9–11^. However, despite initial pilot data showing promise^1,4,5^, three major trials of 2 MHz TCD with or without microbubbles were terminated early due to funding, futility or safety concerns^6,8,12^. In both the NOR-SASS trial and CLOTBUST-ER trial, the investigators identified inadequate thrombus insonation as a reason for the poor outcomes observed^6,8^. Sub-megahertz ultrasound provides consistent insonation of the occluded vessels^13–15^ addressing a limitation of the TCD approach^6^. Sub-megahertz sonothrombolysis was evaluated in the TRUMBI trial which was stopped for safety concerns after 13/14 patients receiving transcranial sonothrombolysis showed bleeding on MRI due to standing wave generation^13,16,17^. Several sub-megahertz sonothrombolysis approaches have been proposed and tested to limit the production of intracranial standing waves and improve the safety profile^15,18,19^. Further work is needed to ensure the safety and efficacy of transcranial sub-megahertz sonothrombolysis.

Hitchcock et al. designed an intermittent continuous wave 220 kHz ultrasound therapy (50 s on, 30 s off) which has been used successfully with exogenous cavitation nuclei in vitro to enhance recombinant tissue plasminogen activator (rt-PA) thrombolysis with sustained stable cavitation^15,20,21^. Using a highly retracted human clot model in vitro, Bader et al.^22^ and Shekhar et al.^20^ observed that rt-PA thrombolysis was not enhanced by sonothrombolysis (exposure to 120 kHz intermittent continuous wave ultrasound), but was enhanced with the addition of Definity, an ultrasound contrast agent (UCA), to the sonothrombolysis treatment arm. Thus, nucleating bubble activity with an echocontrast agent enhanced sonothrombolysis in vitro using this pulsing scheme, but has not been validated in vivo.

The limited predictive value of in vitro studies may be related to the choice of thrombolytic metric. In particular, the correlation between clot mass loss (CML), a quantitative metric for in vitro thrombolysis^23^, and tissue reperfusion measured in vivo is unknown, and the relationship is poorly understood^24^. We performed in vitro studies of sonothrombolysis with Definity in a flow phantom to compare flow measurements with CML as metrics of thrombolytic efficacy^25–27^. Thrombolysis was assessed for two different microcatheter designs in vitro using both clot mass loss and flow as metrics. Additionally, we developed a large animal model that simulates multiple human anatomical, physiological, and biochemical phenotypes.

Culp et al.^28^ described a swine arterial occlusion model based on embolization of a fresh autologous porcine clot into the ascending pharyngeal artery (APA). Subsequently, the effect of 1 MHz pulsed ultrasound and microbubble infusion on rt-PA thrombolysis was investigated in that model^24,28–30^. Recent studies have shown that human clots exhibit greater lysis than porcine clots when exposed to rt-PA (a recombinant human enzyme) and human plasmin^31,32^. Note that the presence of the *rete mirabile* in the porcine cranial circulation prevents catheterization of the middle cerebral artery yet also serves as a trap for exogenously deployed clots. Therefore, the porcine thromboembolism model described by Culp et al.^28^ was adapted using highly retracted human clots instead of fresh autologous porcine clots. We chose to study highly retracted human clots to mimic the fibrinolytic susceptibility of the thrombi responsible for clinical strokes in humans^25,33,34^. Our model also employed clots with physical dimensions (> 14 mm length) that render them recalcitrant to treatment with rt-PA, which accentuates improvements in reperfusion afforded by adjuvant therapies^34^. This porcine xenographic embolism model was used to assess sonothrombolysis with Definity at 220 kHz as a treatment for cranial vascular occlusion, but without ischemia typical of stroke. We hypothesized that intermittent continuous wave 220 kHz ultrasound and Definity enhances rt-PA lysis of human retracted clots and improve recanalization of occluded vessels in vivo, which would correlate to flow measurements in vitro.

The study was performed in two phases both in vitro and in vivo. In the first phase, the highly retracted human clot was deployed directly into the APA and rt-PA was delivered through an end-hole microcatheter at the face of the clot. In the second phase of the study, the external carotid artery was occluded with coils and the clot was deployed from an endovascular delivery catheter stationed in the common carotid and lodged in the APA, proximal to the *rete mirabile*, a tangle of vessels embedded in the skull base. In the second phase of the study, rt-PA was delivered through a multi-side-hole microcatheter traversing the clot axially. Recanalization in the porcine thromboembolism model was assessed via standard clinical angiographic scoring (modified treatment in cerebral ischemia or mTICI^35^) by a neuroradiologist (TAA) blinded to the treatment conditions.

## Results

### In vitro flow phantom

**Figure 1:**
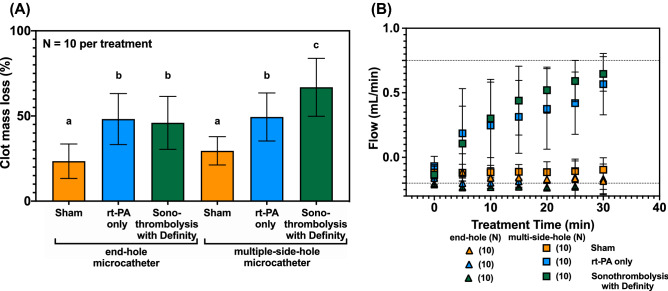
In vitro percent clot mass loss (**A**) and flow (**B**) for human whole blood clots with *n* = 10 for each treatment through each microcatheter. The end-hole microcatheter was positioned at the proximal face of the clot, and the multiple-side-hole microcatheter crossed the clot axially. Flow measurements spanned -0.2 mL/min for a fully occluded APA mimic (retrograde flow due to the treatment infusion) to 0.75 mL for a fully recanalized APA mimic (dotted lines). A one-way ANOVA was performed and significance was shown using compact letter display to identify statistically equivalent groups.

When rt-PA was perfused through the end-hole microcatheter in the first phase in vitro studies, CML was 23.5 ± 10.1% for saline infusion only (sham), 48.2 ± 15.0% for rt-PA infusion only (rt-PA), and 46.0 ± 15.5% for rt-PA and Definity infusion with 220 kHz ultrasound exposure (sonothrombolysis with Definity) (Fig. 1A). No significant difference in thrombolytic efficacy was observed between sonothrombolysis with Definity and rt-PA only treatments using the end-hole microcatheter (*p* > 0.999). Treatment with either rt-PA only or sonothrombolysis with Definity resulted in significantly greater CML relative to sham (*p* < 0.01).

CML in the sonothrombolysis with Definity arm was significantly higher with the multi-side-hole microcatheter than with the end-hole microcatheter (*p* < 0.05). In multi-side-hole microcatheter experiments, CML was 29.5 ± 8.3% for sham, 49.4 ± 14.1% for rt-PA only, and 66.9 ± 17.0%, for sonothrombolysis with Definity (Fig. 1A). CML in the sonothrombolysis with Definity arm with the multi-side-hole microcatheter was significantly higher than the corresponding CML in the rt-PA only arm (*p* < 0.05), which was significantly higher than sham CML (*p* < 0.05).

Both sonothrombolysis with Definity and rt-PA only treatment arms resulted in earlier flow restoration when performed through multi-side-hole microcatheters than when performed through end-hole microcatheters (Fig. 1B). Flow in the APA phantom was unchanged for all three treatments administered through an end-hole microcatheter. Flow due to sham treatments with either the multi-side-hole or the end-hole microcatheter was not significantly different (*p* > 0.37). In contrast, both rt-PA only and sonothrombolysis with Definity treatments using a multi-side-hole infusion microcatheter demonstrated partial flow restoration as early as five minutes and complete flow restoration (0.75 mL/min) by 30 min (Fig. 1B). The total ultraharmonic energy detected during sonothrombolysis with Definity treatments was not significantly different for the end-hole and multi-side hole microcatheters (12.1 ± 15.2 × 10^3^ mV^2^ and 12.3 ± 12.4 × 10^3^ mV^2^, respectively) (*p* > 0.77), indicating no substantial difference in cavitation activity.

### Angiographic reperfusion in xenographic porcine thromboembolism model

**Figure 2:**
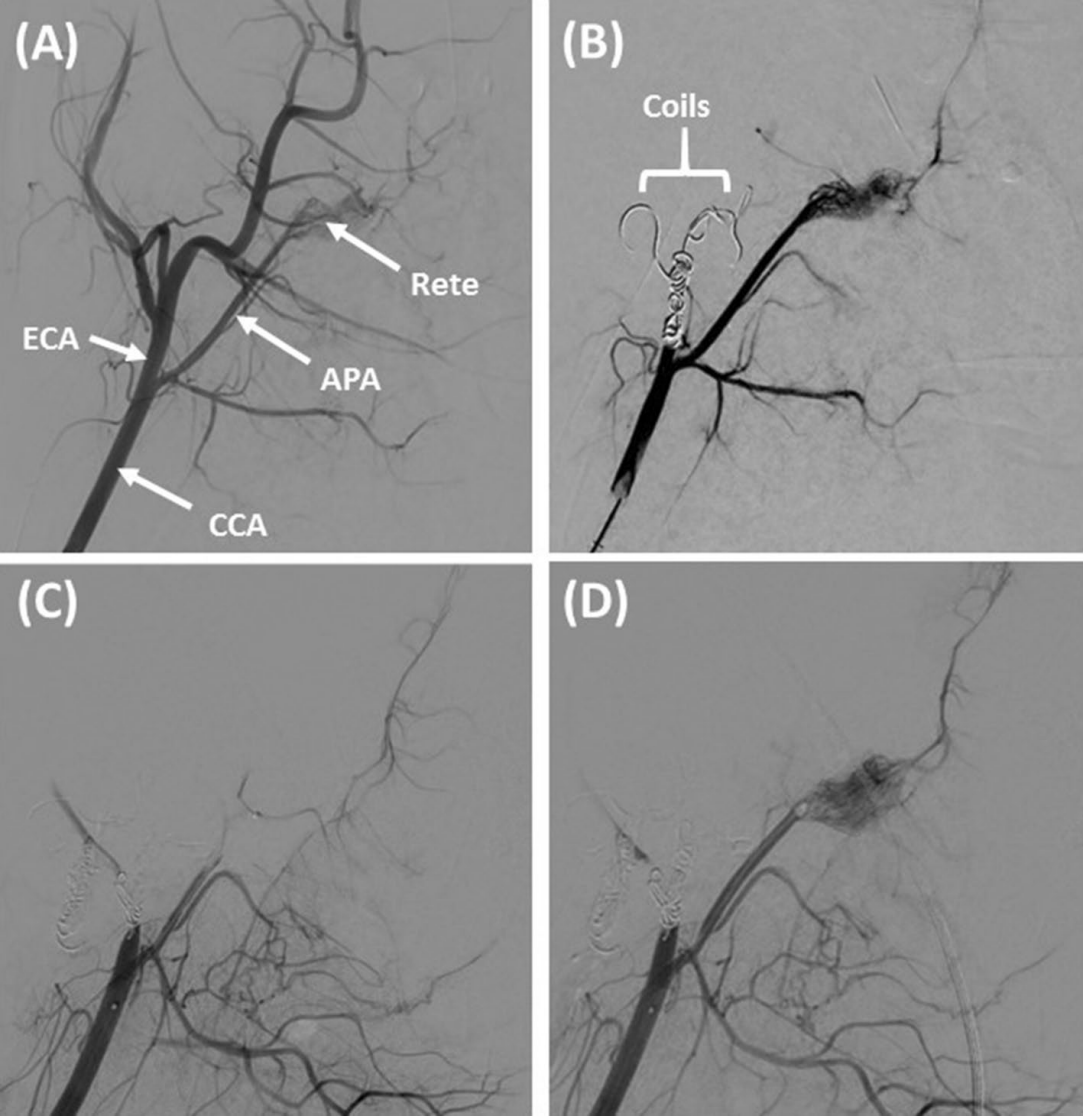
Representative digital subtraction angiographic images during in vivo procedure. Standard anatomy can be observed in (**A**). The common carotid artery (CCA) can be seen branching into the ascending pharyngeal artery (APA) and external carotid artery (ECA). The APA ends in the *rete mirabile* (Rete). The ECA is occluded using bare platinum and hydrogel coils (**B**), which diverts flow preferentially to the APA and reduces collateral circulation to the brain. The APA is successfully embolized with a human whole blood clot (mTICI < 2b) (**C**) and treated for 2 h with either sham, rt-PA only, or sonothrombolysis with Definity (2-h rt-PA only treatment arm shown with mTICI ≥ 2b) (D).

**Figure 3:**
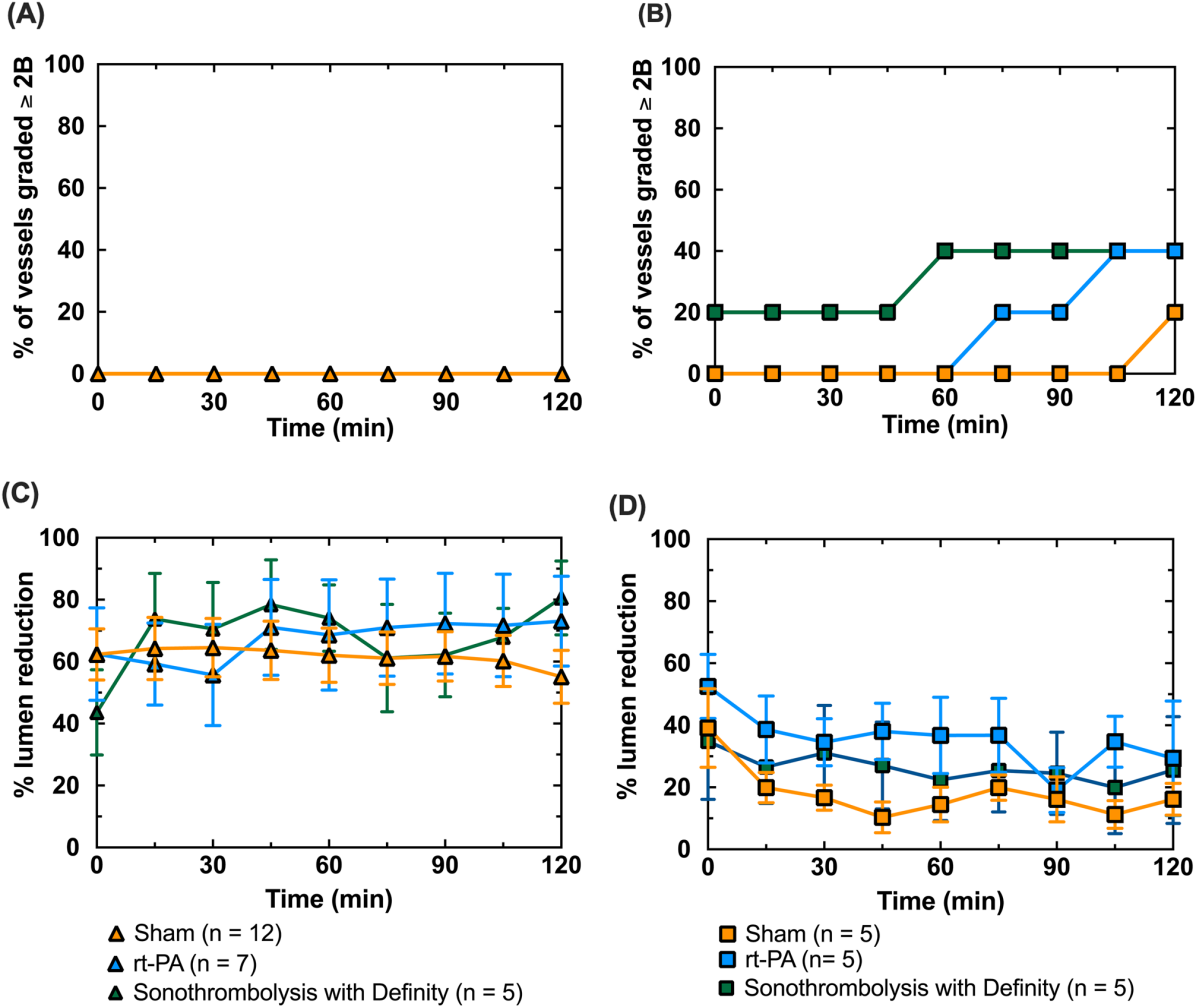
Reperfusion mTICI scores for (**A**) clots treated using the end-hole microcatheter or (**B**) the multiple-side-hole microcatheter. For plot (**A**), *n* = 12 for sham, *n* = 7 for rt-PA only, and *n* = 5 for sonothrombolysis with Definity. In plot (**B**), *n* = 5 for all three treatment arms. No recanalization up to or greater than mTICI = 2b was observed for any clots treated with the end-hole microcatheter (**A**). Reperfusion was accelerated for the rt-PA only treatment arm (75–105 min), and even more for sonothrombolysis with Definity (0–60 min) compared to sham (**B**). Percent APA lumen reduction, was used to quantify vasospasm based on DSA for the end-hole (**C**) and multiple side hole (**D**) microcatheters.

Most porcine APA vessels (39/40) were successfully occluded by transcatheter embolization with retracted human clots and were included in the study (Figs. 2, 3). Following occlusion of the APA, porcine temperatures varied by no more than 1.5%, porcine heart rate by no more than 28% and porcine systolic blood pressure by no more than 27% compared to baseline^36^. Shown in Fig. 3 is the percentage of APA vessels which achieved successful reperfusion (mTICI grade of 2b or 3) as a function of time for each of the three treatments arms (sham, rt-PA only, sonothrombolysis with Definity). When rt-PA was infused through the end-hole microcatheter (phase 1 studies), none of the occluded APA vessels were successfully reperfused during the 120 min treatment period, and no significant difference was found between any of the treatment groups (*p* > 0.99) (Fig. 3A). Five of the 15 clots treated with multi-side-hole microcatheters (phase 2 studies) were successfully reperfused, including 1 sham, 2 rt-PA only, and 2 sonothrombolysis with Definity (Figs. 2D, 3B). One sham achieved successful reperfusion (mTICI ≥ 2b) at 105–120 min, which was significantly longer than rt-PA only (*p* < 0.01) or sonothrombolysis with Definity (*p* < 0.01) treatments (Fig. 3B). Clots treated with rt-PA only achieved reperfusion after 75–105 min of treatment, and sonothrombolysis with Definity treated clots recanalized the most rapidly, between 0–60 min. The degree of recanalization over time was significantly different between all three treatment arms (*p* < 0.05).

### APA Vasospasm

**Figure 4:**
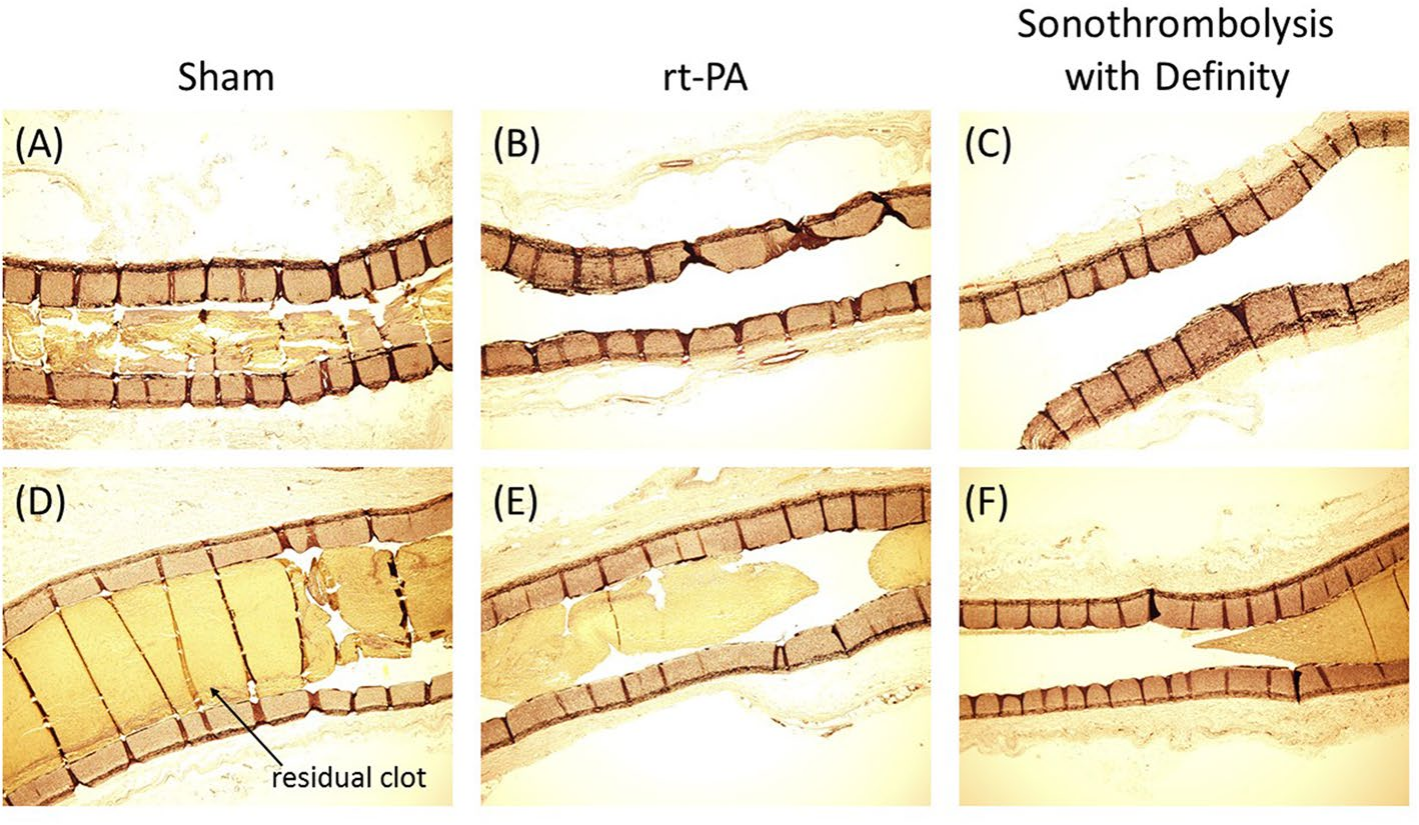
Representative longitudinal histology images of residual clot burden in APAs with Verhoeff-Van Giessen (VVG) stain. Residual clot volume can be seen in vessels in the sham treatment arm (**A**, **D**) rt-PA only treatment arm (**B**, **E**) or the sonothrombolysis with Definity treatment arm. Images **A**–**C** show vessels for which the perfusion was graded as ≥ 2b at 120 min on the mTICI scale and images D-F show vessels graded < 2b at 120 min.

Figure 3C and D shows the percent APA lumen reduction due to vasospasm for each of the treatment arms as a function of time using two different APA embolization paradigms (phases 1 and 2 of the study). In phase 1, severe APA vasospasm (> 50% APA lumen reduction)^37^ was present throughout the 120 min treatment period for most vessels (Fig. 3C). When vasospasm was encountered in phase 2 studies, the vessel relaxed within 30 min of APA occlusion (*p* < 0.05) (Fig. 3D). Representative stained longitudinal APA sections differentiated by treatment are shown in Fig. 4(A-F) with representatives of both vessels which recanalized (4A-C) and vessels which did not recanalize (4D-F).

## Discussion

Our in vitro experiments show that the design and position of the microcatheter used for delivery of Definity is an important modifier of fibrinolytic acceleration. Nonetheless, for sham-treated clots, no difference in CML or flow was observed regardless of microcatheter design and position (*p* > 0.99) (Fig. 1). This finding suggests that the mechanical penetration of the clot with the microcatheter did not accelerate recanalization. Clots treated with rt-PA had a higher CML compared to sham treated clots regardless of microcatheter design or position, consistent with previous in vitro studies (Fig. 1A)^38–41^. On the other hand, sonothrombolysis with Definity improved CML only when the contrast agent was infused directly into the clot through a multi-side-hole microcatheter (Fig. 1A, B) in contrast to previous in vitro studies^1,3,4^. The reduced surface area of the clot exposed to the thrombolytic in our in vitro thrombolytic model could be one explanation for this disparity.

Definity delivered throughout the clot promoted cavitation nucleation and sustained bubble activity, which has also been observed other in vitro studies^15,22^. This pattern suggested that the adjuvant effect of Definity and ultrasound relied on the spatial proximity of bubble activity, which is linked to the diffusion of Definity throughout the clot^40,42–44^. Datta et al. noted that rt-PA penetration into a clot and subsequent activation of plasminogen was enhanced by the presence of stable cavitation nucleated by Definity and sub-megahertz ultrasound^40^. Acconcia et al. observed that microbubbles exposed to 1 MHz ultrasound tunneled over 80 µm into the fibrin network^45^. This distance is small compared to the length of our entire clot, but could enhance the creation of a channel if microbubbles are delivered throughout the clot length^45^. An increase in CML for a particular treatment was not always accompanied by an increase in flow for the same treatment (Fig. 1), possibly due to the low flow velocity or shunting of flow through the external carotid artery (ECA) mimic. This data highlights the disparity of these two metrics of thrombolytic efficacy (clot mass loss *vs*. flow). As flow restoration is the ultimate goal of stroke intervention, flow is a preferable metric for translational studies.

Our APA model was based on the porcine model of vascular occlusion developed by Culp et al.^28^. We modified this approach to accommodate a highly retracted human xenographic clot formed exogenously. However, in our phase 1 study, no reperfusion was observed in any of the three treatment arms. This lack of rt-PA efficacy was likely due to vasospasm, which would obstruct or retard flow regardless of the degree of lysis. Vasospasm would have hampered the flow of rt-PA, delaying or preventing lysis entirely. Additionally, grading perfusion with the mTICI scale was complicated by vasospasm (Fig. 3C). Vasospasm may be a contributing factor to poor perfusion outcomes after intervention and may also be a target for therapy using ultrasound targeted drug delivery in future studies^46^.

To deploy the clot percutaneously into the APA without provoking severe vasospasm (Fig. 3C), we performed APA embolization from a catheter stationed in the common carotid artery (CCA) (Fig. 2B). Initial occlusion of the ECA by coil embolization facilitated targeted delivery of clot into the APA, a vessel that is much smaller than the ECA. The ECA is a major source of collateral blood flow to the brain when the APA is occluded (Fig. 2A). In our model, coil embolization of the ECA places the burden for anterograde *rete mirabile* perfusion exclusively on the APA, removing competitive flow that might otherwise complicate assessment of angiographic reperfusion according to the mTICI scale. Thus, the advantages of ECA occlusion in our model are multifold.

Following occlusion of the ECA, delivery of the clot into the common carotid led to rapid and complete occlusion of the APA. Despite no attempt to control for various blood types and antigens, human xenographic clots did not cause any obvious distress to the pigs based on the measured vitals^36^. However, the effects of human whole blood clots in the porcine vasculature over longer periods (greater than 8 h) are unclear. Additionally, the highly retracted, fibrin-dominant exterior of the clots may limit the presentation of foreign antigens in the porcine blood, reducing the incidence of immunologic reactions^31,47,48^. The methylprednisolone and carprofen, given to prevent porcine hypersensitivity to Definity, may also have suppressed the immune response to the human whole blood clots.

Based on the mTICI scores, we observed an increase in perfusion of the vascular territory downstream from the APA during the two-hour treatment period for all three treatment arms in phase 2 (Fig. 3B). The adjuvant effect of Definity and 220 kHz continuous wave ultrasound exposure was most prominent early in the treatment process. Adjuvant Definity plus ultrasound increased the percentage of occluded vessels achieving successful reperfusion in the first 60 min of treatment by 20% and reduced the minimum time to successful reperfusion with rt-PA only by 45 min. Khatri et al.^49^ showed that the probability of a good clinical outcome is increased by 10% for every 30-min reduction in the time to successful angiographic reperfusion (TICI 2 or 3) during treatment with transcatheter intra-arterial rt-PA. Consequently, the quantitative acceleration of successful reperfusion observed in our study has the potential to translate into improved clinical outcomes.

An improvement in mTICI scores for 20% of sham treated clots was noted in the final 15 min of treatment (Fig. 2B). Culp et al.^28,29^ observed similar results for saline treated porcine thrombi. In vivo, spontaneous complete recanalization without any treatment has been observed in clinical trials such as the PROACT II trial (18–30%), but was not observed in our study^50^.

The design and position of the microcatheter used for delivery of Definity was an important modifier of fibrinolytic acceleration in our in vivo studies. Overall, two thirds of the vessels treated with multi-side-hole microcatheters did not successfully reperfuse regardless of the therapy arm. Conversely, none of the vessels treated with the end-hole microcatheter were successfully reperfused, regardless of the therapy arm. Vasospasm was an important factor associated with reperfusion failure, particularly in phase 1 of our study (Fig. 2C). Vasospasm likely inhibited angiographic reperfusion, as constriction of the APA around the intraluminal blood clot prevented downstream flow, even if the intraluminal blood clot were enzymatically degraded. Nonetheless, our in vitro study suggests that even without vasospasm, delivery of rt-PA through an end-hole microcatheter at the proximal face of an occlusive clot does not effectively achieve successful reperfusion (Fig. 1). This is consistent with the results of prior clinical trials that employed a similar treatment strategy^51^.

Our experimental design was intended to disadvantage treatment with rt-PA only, so that the differential benefit provided by adjuvant treatment with Definity and ultrasound could be quantified. Toward that end, our study employed clots with a length of 1.5 cm. Such clots are known to be resistant to treatment with intravenous (IV) rt-PA^34^. Additionally these clots are older and larger than the 2–6 h old autologous venous clots used by Gao et al. or Culp et al. in a similar porcine APA model, although important to note that these studies were not investigating the effects of rt-PA mediated thrombolysis, only ultrasound and microbubbles^24,28,29^. These fresh autologous clots are softer and more prone to lysis even in the absence of rt-PA^48^. Brown et al. and Florres et al. used human clots in a rabbit model, which were much smaller (1.0 mm sections in both), but assessed infarct volume rather than recanalization^23,52,53^. In addition to the size and degree of retraction affecting lytic resistance, the clot location could also have contributed to this high percentage of clots which failed to reperfuse. The porcine cranial circulation has extensive collaterals, some of which can only be observed on angiography after the primary vessel is occluded. One explanation for the low degree of reperfusion in our model was slow exchange of plasminogen between circulating blood and the stagnation zone at the proximal face of experimental clots. Prolonged stasis of flow in this region was confirmed angiographically in our study. The direct effect of Definity on the enzymatic activity of rt-PA was not examined in this study as microbubbles can affect the measurement of rt-PA using spectrophotometric assays^54–56^. However, Hitchcock et al. noted that Definity and rt-PA did not significantly effect thrombolysis in the absence of ultrasound compared to rt-PA alone^21^.

The in vitro flow phantom had several important differences in relation to our porcine thromboembolism model. The in vitro system utilized continuous flow, rather than physiological pulsatile flow. Our continuous flow velocity was 4 cm/s based on the plug flow assumption, which is in the range of velocities of partially occluded middle cerebral arteries in humans^57^. However, flow velocity can vary widely during recanalization and pulsatile flow can reach peak velocities greater than 100 cm/s in a patent middle cerebral artery^57^. Also, vasospasm was lacking in the flow model, which was assessed in vivo.

The porcine cranial thromboembolism model is not representative of ischemic stroke as the porcine cranial collateral circulation is more robust than the human cranial circulation^58^. The porcine cerebral anatomy is defined by a network of vessels and capillaries including the *rete mirabile,* which prevents access to the Circle of Willis and cerebral arteries using intravascular catheters^58^. Therefore, an occlusion was only established upstream of the *rete mirabile.* To translate benefits to the ischemic brain, further translational studies are needed to verify the effects of sonothrombolysis with Definity on reperfusion and long-term outcomes in a large animal ischemic model. Despite these limitations, investigation of a sub-megahertz approach has potential to obviate the need for careful alignment of the ultrasound field with the ischemic occlusion^6,8,12,16,59^.

Current stroke therapy includes IV thrombolytic infusion and mechanical embolectomy based on thrombus location and resource availability^60^. Intra-arterial thrombolytic therapy has largely been replaced by mechanical thrombectomy, although the need exists for development of treatment strategies for thrombi resistant to lytics or mechanical thrombectomy^61,62^. Further research in this model should be performed to assess the thrombolytic efficacy of intravenous Definity and rt-PA in our retracted human clot model in vivo.

## Conclusions

This study demonstrates that concurrent administration of Definity and 220 kHz ultrasound significantly accelerates resolution of thromboembolic vessel occlusions treated with rt-PA in vitro and in vivo. Using widely accepted clinical criteria for technically successful reperfusion, rt-PA concurrent with Definity and 220 kHz ultrasound reduced the time to successful angiographic reperfusion in vivo by 45 min, a time savings with potential for clinically significant positive impact. Thus, we accepted our hypothesis that intermittent continuous wave 220 kHz ultrasound and Definity enhances rt-PA lysis of human retracted clots and accelerates recanalization of occluded vessels. Our study further shows that the relationship between the reduction of clot burden (CML) and the restoration of flow is complex and suggests that delivery of a thrombolytic at the surface of a clot is insufficient to effect meaningful thrombolysis. Further development should focus on methods to improve flow based on improved penetration of thrombolytics throughout a clot. Given that clinically proven modern paradigms of stroke therapy are based on mechanical revascularization of large vessel occlusions and administration of IV rt-PA, further study is needed to understand how adjuvant sub-megahertz ultrasound therapies can be used to improve therapeutic outcomes.

## Materials and methods

### Retracted human whole blood clot synthesis

Human whole blood was obtained from donors under an approved IRB protocol (# 2012–2575) by the Institutional Review Board at the University of Cincinnati in accordance with all relevant guidelines and regulations and with informed consent. Clots were prepared following the protocol established by Sutton et al.^47^. One milliliter aliquots of fresh human whole blood were pipetted into borosilicate glass Pasteur pipettes (inner diameter, 4 mm, 4 inches long, Chang Bioscience, Fremont, CA, USA) and the bottom was sealed with beeswax. The aliquots were first incubated at 37 °C for 3 h, and then refrigerated at 4 °C for 3–17 days to promote the formation of highly retracted clots^47^. Lytic susceptibility of whole blood clots formed in this way do not change significantly within this time period^63^. Prior to use, clots were removed from the glass pipettes, trimmed to 1.5 cm lengths, and the width of each clot was recorded using a dissection microscope (BX51, Olympus, Melville, NY). A 1.5 cm length was chosen to evaluate the type of highly retracted thrombi that do not respond well to conventional lytic therapy^34^. For in vitro studies, each clot was also blotted and weighed following a previously established protocol^38^. Clots were loaded into 1 mL syringes of 50% Optiray (Liebel-Flarshiem LLC, Raleigh, NC, USA): 50% phosphate buffered saline (PBS) for delivery through an 8 F guide catheter (Vista Brite Tip, Cordis, Santa Clara, CA, USA).

### In vitro* flow phantom thromboembolism protocol*

**Figure 5:**
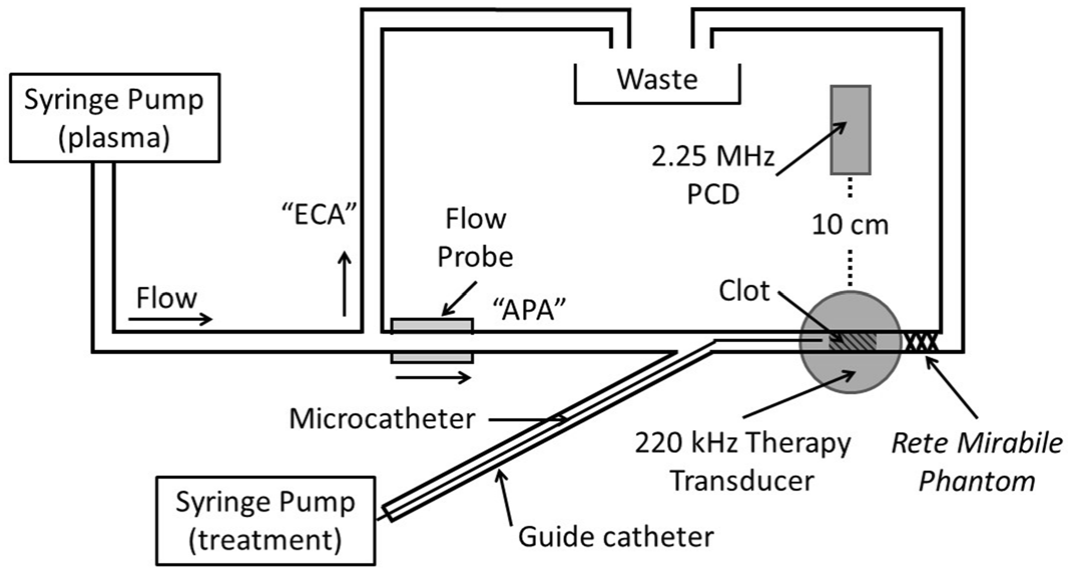
Schematic of in vitro vascular occlusion model. A highly retracted human whole blood clot was deployed in a phantom of the ascending pharyngeal artery (APA) via an 8 F guide catheter and held in place by a *rete* phantom. A collateral tube simulated flow in the external carotid artery (ECA). A flow probe monitored flow through the occluded APA phantom. A passive cavitation detector (PCD) was aligned with the clot and field of the 220 kHz transducer for sonothrombolysis nucleated by Definity.

A porcine carotid artery flow phantom was created in a 37 °C water tank to direct flow to two branching tubes, one mimicking the ascending pharyngeal artery (APA), another the external carotid artery (ECA) (Fig. 5). Latex tubing (2.4 mm inner diameter) primed with sodium-citrate anticoagulated porcine plasma (Lampire Biological Laboratories, Pipersville, PA, USA) was pumped through the flow phantom with a syringe pump (Pump 11 Elite, Harvard Apparatus, Holliston, MA) at a rate of 1.5 mL/min. This flow rate was selected to be within the range of flow rates observed by Alexandrov et al.^57^ in partially occluded cerebral vessels. A Luer connector with a metal mesh mimicked the *rete mirabile*^58,64^. An 8 F guide catheter was passed through a hemostasis valve (AccessPLUS, Merit Medical, South Jordan, UT, USA) to deliver clots into the APA mimic and provide microcatheter access to the flow phantom (Fig. 5). A flow probe (ME1PXN, Transonic, Ithaca, NY, USA) was placed 20 cm proximal to the simulated *rete mirabile*. A preformed clot was embolized into the APA phantom at the location of the *rete* mimic using the guide catheter.

A custom-designed unfocused transducer (220 kHz center frequency, 38 mm aperture) was positioned so that the clot in the vessel phantom was in the natural focus (5.2 cm). An intermittent continuous wave ultrasound insonation scheme (50 s on, 30 s off) was employed (220 kHz, 0.44 MPa peak-to-peak) for clots treated with rt-PA and Definity^15,22^. The transducer was driven with a function generator (33250A, Agilent Technologies, Inc., Santa Clara, CA) and power amplifier (Model 75A250, Amplifier Research, Souderton, PA). Cavitation signals were monitored using a passive cavitation detector (19 mm aperture, 2.25 MHz center frequency, 595516C, Picker International, Cleveland, Ohio, USA) aligned following the protocol described by Bader et al.^22^. The received RF signal was filtered with a 10 MHz low-pass filter (J73E, TTE Inc, Los Angeles, CA, USA) and amplified using a wideband low-noise amplifier (CLC100, Cadeka Microcircuits LLC, Colorado, USA). The signal was digitized (12-bit resolution, 10 ms duration, 31.25 MHz sampling frequency) using a digital oscilloscope (Picoscope 4227, PicoTech, St. Neots, Cambridgeshire, UK). Total cavitation energy was quantified from the power spectrum calculated over 10 ms windows captured every 13 ms and summed over the full 30 min treatment, following the method described by Bader et al.^22^.

Two types of microcatheters were used, one with a single end hole (2.8 F Rapidtransit, Codman Neurovascular, Raynham, MA, USA), and the other with multiple side hole positioned 5 cm along the distal end (2.9 F Micromewi, 5 cm infusion length, Medtronics, Minneapolis, MN, USA). Each microcatheter was deployed through the guide catheter and either positioned at the face of the clot (end hole), or advanced across the clot (multi-side hole) based on the in vivo protocols in phase 1 and 2, respectively. Saline, 1 mg/mL rt-PA only, or 1 mg/mL rt-PA mixed with 2 μL/mL Definity was infused through the microcatheters for 30 minutes^15^ at a rate of 10 mL/hr (*n* = 10 per treatment arm, statistical power > 90%) for the sham, rt-PA only, and sonothrombolysis with Definity treatment arms, respectively. The difference between the initial and final clot mass (CML) was calculated as a percentage of the initial mass.

### Porcine xenographic thromboembolism model

The porcine thromboembolism research protocol was approved by the University of Cincinnati Institutional Animal Care and Use Committee and all procedures were performed in accordance with its specified guidelines and the ARRIVE guidelines^65^. Heart rate (Surgivet V9203, Smith Medical, Oakdale, MN, USA) and rectal temperature (Surgivet V9203) were monitored continuously and recorded at 5-min intervals throughout the procedure. Blood pressure was monitored (Surgivet V9203) before and after clot deployment into the APA and intermittently throughout the procedure by opening the stopcock to the femoral sheath. Two clot deployment methods were employed in different phases of the study. In phase 1, an occlusion was established by embolizing retracted human clots directly into the APA through an 8 F guide catheter stationed in the APA. Treatment was administered through end-hole microcatheters stationed at the face of the occlusion. Phase 2 experiments mitigated APA vasospasm noted in the former approach by avoiding placement of the embolizing guide catheter into the APA. For this method, the ECA was first occluded by coil embolization, and the embolizing guide catheter was stationed in the common carotid artery (CCA). Occlusion of the ECA in this way ensured that human clots embolized from a guide catheter stationed in the CCA would enter the target APA, rather than the larger and higher flow ECA. After embolizing a retracted human clot into the APA, a multi-side-hole microcatheter was advanced across the clot and used to administer treatment directly into the clot. A total of 12 swine (Yorkshire, mean weight 49.1 ± 4.6 kg) and 8 swine (Yorkshire, mean weight of 52.6 + 3.2 kg ) were used in phase 1 and phase 2 respectively. A power analysis was performed based on the odds ratio and found n = 5 provided a power level between 70–80% comparing all treatment groups. Inclusion of each pig was based on successful establishment of a clot in the APA during embolization. Treatment was randomized by coin flip after establishing an occlusion in the APA but was not blinded as the interventionalist also set up, aligned, and monitored the ultrasound therapy. Each pig was treated bilaterally for a total of 24 occluded vessels in phase 1: 12 sham, 7 rt-PA only, and 5 sonothrombolysis with Definity. A total of 15 APA vessels were occluded in phase 2: 5 in each treatment arm.

### Anesthetic care

Swine were sedated using IM acepromazine (1.1 mg/kg) and ketamine (33 mg/kg) and put on assisted ventilation with continuous isoflurane (2–3%). To avoid adverse reactions to Definity, pigs were pre-treated with 40 mg of IV methylprednisolone and 2 mg/kg carprofen^28,36^. Intravenous (IV) nicardipine (0.1 mg/kg/hr) was also given to prevent vasospasm during and after embolization^66^.

### Angiographic technique and measurements

10 F introducer sheaths were placed in both femoral arteries using the Seldinger technique^67^. The CCA’s were catheterized bilaterally using 8 F guide catheters (Vista Brite Tip). Digital subtraction angiography (DSA) was performed with a FD20-Allura-Clarity angiographic system (Philips, Best, The Netherlands). The luminal diameter of each APA and ECA was measured on DSA images using the digital image processing software of the angiographic system (Fig. 2A). Percent lumen reduction of the APA was calculated as $\frac{D\left({t}_{i}\right)-D\left(t\right)}{D\left({t}_{i}\right)} 100\%$, where $D\left({t}_{i}\right)$ is the initial diameter prior to embolization and $D\left(t\right)$ is the diameter at each time point, *t.*

### Coil embolization of ECA

To enable embolization of retracted human clots directly into the porcine APA, the embolizing guide catheter was advanced into the proximal APA in phase 1. In phase 2, the embolizing guide catheter was stationed in the CCA, approximately 4 cm proximal to the CCA bifurcation, and the ECA was occluded by fluoroscopically guided embolization using a combination of bare platinum pushable coils, detachable Hydrocoils (Microvention Inc, Aliso Viejo, CA, USA) and Azur 35D coils (Terumo, Somerset, NJ, USA). Coil embolization of the ECA was performed bilaterally through 5 F Berenstein catheters (Merit Medical), coaxially advanced from the 8 F embolizing guide catheters. Twenty minutes after coil embolization of the ECA, DSA was performed through the 8 F guide catheter stationed in the CCA (Fig. 2B).

### Embolization of porcine APA with retracted human clots

To perform APA embolization, a clot was transferred to the 8 F embolizing guide catheter. Transfer was facilitated using a large bore one-way stopcock (G00164, Cook Medical, Bloomington, IN, USA). Fluoroscopically guided embolization of the clot into the APA was performed by slow and steady injection of contrast media into the guide catheter containing the clot. Following each APA embolization, sequential DSA sequences were acquired, by injection of contrast media through the guide catheter, to assess the degree of APA occlusion and distal perfusion of *rete mirabile* using the modified Thrombolysis In Cerebral Infarction (mTICI) scale^35^.

### Transcatheter treatment of APA clots

For treatment administration, the end-hole microcatheter was advanced through the 8 F guide catheter to the proximal edge of the APA clot (within 5 mm), or the multi-side-hole microcatheter was advanced across the APA clot, from the coaxial 8 F guide catheter. The microcatheter was infused over 2 h with either 20 mL normal saline (sham), 20 mL rt-PA (1 mg/mL) only, or 20 mL rt-PA (1 mg/mL) plus Definity (2 μL/mL) (sonothrombolysis with Definity). The insonation parameters were the same as those described for the in vitro studies above. A cone beam CT scan of each porcine head was performed before bilateral ECA embolization (XperCT, Philips Healthcare, Best, Netherlands) and integrated needle guidance software was employed to position the ultrasound transducer (XperGuide 3D tools, Philips).

### Angiographic assessment of target vessel recanalization and distal reperfusion

Sequential DSAs were obtained every 15 min during the 2-h treatment period by injecting 6–8 mL of undiluted Optiray down each guide catheter. At the conclusion of each experiment, the pig was euthanized and decapitated. APA recanalization and *rete mirabile* perfusion at each time point was graded on the mTICI scale by an interventional neuroradiologist (TAA) blinded to the treatment with mTICI < 2b (Fig. 2C) and mTICI ≥ 2b (Fig. 2D). An unblinded mTICI score was determined during the procedure by a trainee (RTK). Grading disagreements were settled according to a secondary review conducted by both readers concurrently, without unblinding of the first reader. The reported mTICI score represents the consensus mTICI score agreed upon by the two readers.

### Histology of APA and associated clot

Each APA was dissected following the protocol developed by Eliyas et al.^64^. Using a 30 G needle and syringe, each APA was pressurized to 90 mmHg with 10% formalin. Formalin fixed APA specimens were embedded in paraffin, cut longitudinally along the vessel and stained with Verhoeff-Van Gieson stain.

### Statistics

A one-way ANOVA with a Tukey honest significant difference (HSD) test was used to assess CML differences between treatment arms for each microcatheter design. A linear mixed model was used to compare the effect of each microcatheter design and treatment on flow as a function of time in vitro and on vasospasm as a function of time in vivo. The mTICI scores were dichotomized into failure (mTICI < 2b), or success (mTICI ≥ 2b). The resultant data was fit to a logistic regression model for pairwise comparison between each of the three treatment arms. All statistical computations were done in R (v. 2.14.1, R Foundation for Statistical Computing, Vienna, Austria). A *p* value < 0.05 was considered statistically significant. Power analysis of the in vitro data was computed based on an effect size of 0.779 calculated from the data and the statistical tables in published by Cohen^68^. Power analysis for the in vivo study was computed using an algorithm for logistic regression power developed by Demidenko^69,70^.

## Data Availability

The data sets generated and analyzed during the current study are available from the corresponding author on reasonable request.
